# Prenatal diagnosis and postnatal course in four fetuses with very rare pulmonary artery anomalies

**DOI:** 10.4274/tjod.galenos.2021.40035

**Published:** 2021-03-12

**Authors:** Reyhan Ayaz, Oya Demirci, Özgür Aydın Tosun, Öykü Tosun

**Affiliations:** 1 İstanbul Medeniyet University Faculty of Medicine, Department of Perinatology, İstanbul, Turkey; 2 Zeynep Kamil Maternity and Children Training and Research Hospital, Clinic of Perinatology, İstanbul, Turkey; 3 İstanbul Medeniyet University Faculty of Medicine, Department of Obstetrics and Gynecology, İstanbul, Turkey; 4 İstanbul Medeniyet University Faculty of Medicine, Department of Pediatric Cardiology, İstanbul, Turkey

**Keywords:** Agenesis of ductus arteriosus, LPA originating from the ascending aorta, Absent Pulmonary Valve syndrome, LPA sling, right aortic arch, tetralogy of Fallot

## Abstract

Pulmonary artery (PA) anomalies are very rare congenital cardiac malformations, a significant number of which remain unrecognized or misdiagnosed during the prenatal period. We report the prenatal diagnosis and outcome of pregnancy with fetal PA anomalies and discuss the related management issues. We identified four cases of prenatally diagnosed rare PA anomalies that were seen and confirmed in the newborn period by echocardiography and computed tomographic angiography at our center from 2018 to 2020. The course of the pregnancy, perinatal outcome, and the postnatal course in each case were analyzed. Three fetuses were born by repeat cesarean section approximately at 39 weeks of gestation and the other woman delivered vaginally. Of the abnormal origin of the left PA (LPA) in two patients, the first had right PA abnormalities derivating from the ascending aorta, and in the second, the LPA originated from the right PA. Two patients had agenesis of ductus arteriosus (DA), the first was accompanied with tetralogy of Fallot (TOF) and right aortic arch with a normal pulmonary valve, the second patient presented with an Absent Pulmonary Valve syndrome with TOF. Prenatal ultrasonography can be used to correctly diagnose the abnormal origin of the PA branches. Branching of the PA, presence of DA, location of the aortic, and ductal arch by the trachea should be routinely screened in the prenatal anatomic examination and the three-vessel and trachea view can determine the primary clues of PA malformations.

## Introduction

Congenital pulmonary artery (PA) anomalies are extremely rare among cardiovascular anomalies^(1)^. These pathologies usually present with other complex cardiac diseases, when these anomalies are isolated, they are likely to go unrecognized during fetal life. Accurate prenatal diagnosis of congenital heart disease prevents neonatal morbidity and mortality and provides appropriate preoperative conditions, and improves surgical outcomes^(2)^. We describe four cases including left PA (LPA) sling, LPA originating from the ascending aorta (AOLPA/hemitruncus), and two cases of agenesis of ductus arteriosus (DA). Patients with DA agenesis had accompanying tetralogy of Fallot (TOF) - absent pulmonary valve (APVS) and TOF - right aortic arch (RAA), respectively. LPA sling, also known as aberrant LPA, derives from the superior and posterior aspect of the right PA instead of the main PA and then turns sharply leftward posterior to the trachea to enter the hilum of the left lung^(3)^. An LPA originating from the ascending aorta is characterized by the anomalous origin of left PAs from the posterolateral wall of the ascending aorta in the presence of two semilunar valves^(4)^. The DA is a unique vessel of fetal circulation that connects the pulmonary trunk and descending aorta. The underlying cause of DA agenesis is not well known and it is associated with other cardiac anomalies such as TOF, absent pulmonary valve, truncus arteriosus, and ventricular septal defect^(5)^. The aim of this study was to review our experience of prenatal ultrasonographic features of PA abnormalities. We emphasize that the three-vessel and trachea (3VT) view, which is the most important view in the diagnosis of PA anomalies, and in addition, LPA originating from the ascending aorta should be taken into consideration in the differential diagnosis when the fourth vessel is detected in the 3VT.

## Materials and Methods

A total of 6752 women with singleton pregnancies from 18 to 30 weeks gestation were examined from September 2018 to February 2020 at the Department of Perinatology and Pediatric Cardiology, Istanbul Medeniyet University, Istanbul, Turkey. Fetal anatomic and echocardiographic examinations were performed using a Voluson E6 Expert ultrasound device, Samsung Ultrasound H60, and the Esaote MyLab™9 Platform. All patients underwent a complete fetal anatomic screening according to the International Society of Ultrasound in Obstetrics & Gynecology practice guideline. The standard four chambers and ventricular outflow tracts of the fetal heart were obtained routinely during the fetal anatomic screening for all patients in detailed two-dimensional, color Doppler echocardiography. After finding a cardiac anomaly, detailed echocardiographic examinations including 3VT, aortic and ductal arch, PA branches, aortic long axis, superior vena cava, inferior vena cava inflow, pulmonary venous return were assessed. We used color Doppler blood flow to track the PA branches and DA. Chromosome analysis and fluorescent *in situ* hybridization (FISH) analysis for microdeletion 22q11.2 were recommended to all patients. In addition to ultrasonography, demographic features and medical histories of all the mothers were reviewed and the perinatal outcomes were recorded. In the postnatal period, to verify the prenatal diagnosis of all PA anomalies different imaging modalities, such as echocardiography, cardiac computed tomography (CT) angiography, and angiography were used. The study was approved by the hospital ethics committee and all of the pregnant women with PA anomalies provided written informed consent.

## Results

**Figure 1:**
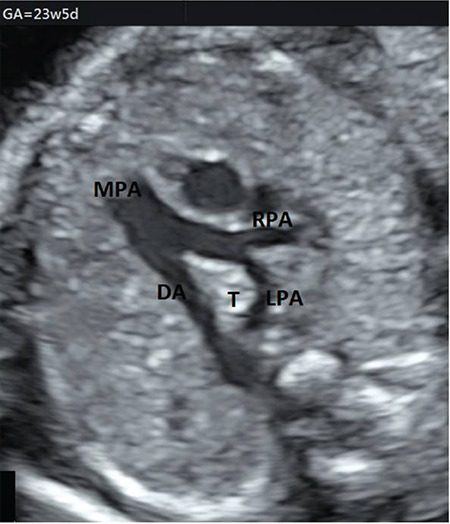
The LPA originates from the right pulmonary artery LPA: Left pulmonary artery

**Figure 2:**
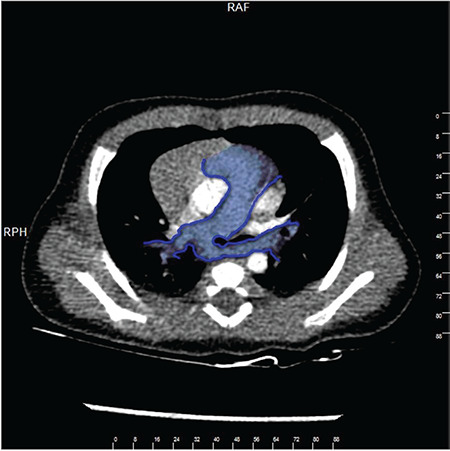
Axial contrast-enhanced computed tomography confirmed the aberrant LPA LPA: Left pulmonary artery

**Figure 3:**
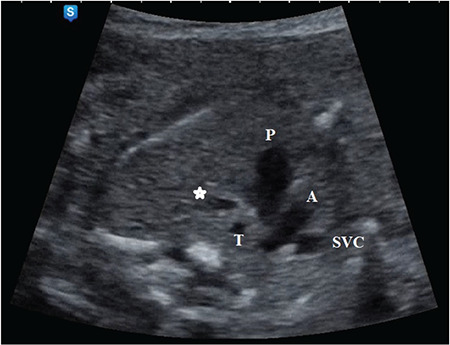
The aortic arch and ductal arch were located on the right side of the trachea, and four vessels were seen on the 3VT view P: Main pulmonary artery, A: Aorta, SVC: Superior vena cava, T: Trachea, *: LPA, LPA: Left pulmonary artery

**Figure 4:**
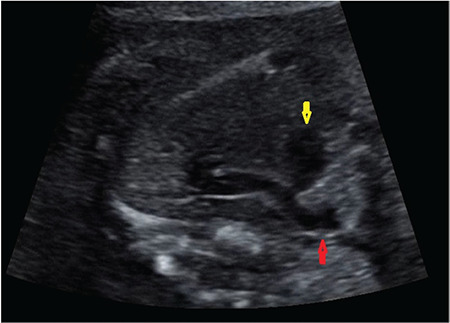
The LPA originated from the posterior aspect of ascending aorta and then turning left, crossed midline anterior the trachea towards the hilum of the left lung Yellow arrow: Pulmonary artery, Red arrow: Aolpa, LPA: Left pulmonary artery

**Figure 5:**
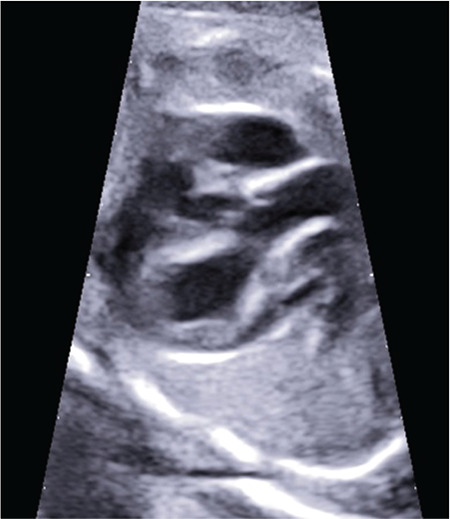
The right aortic arch, malalignment ventricular septal defect, overriding aorta in the five-chamber view were shown

**Figure 6:**
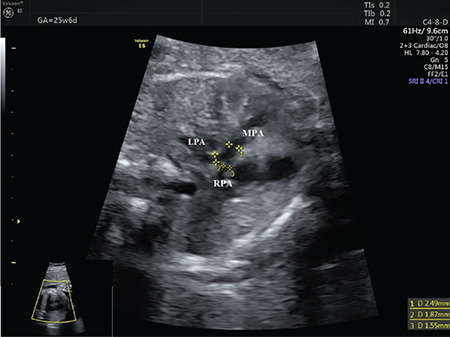
The diameters of the pulmonary annulus, right and LPA were seen on the 3VT view LPA: Left pulmonary artery

**Figure 7:**
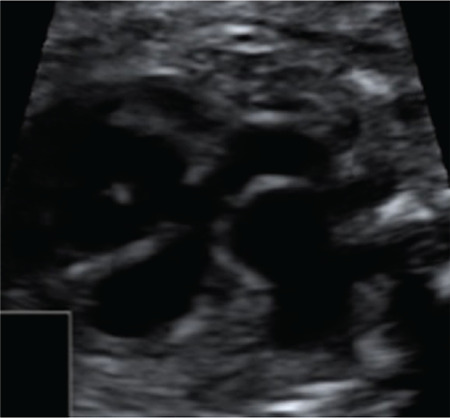
Hypoplasia of the pulmonary annulus, subaortic ventricular septal defect with the overriding aorta, severe dilatation of the pulmonary trunk were seen on the outflow tract of the right ventricular view

**Figure 8:**
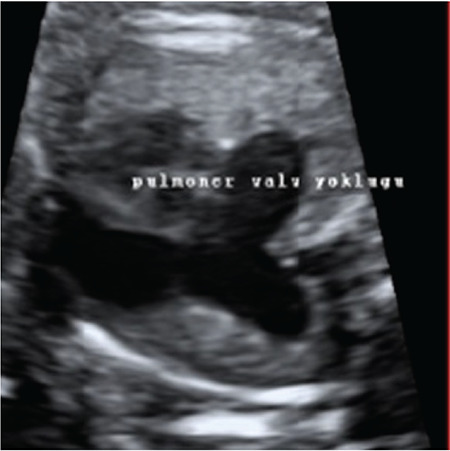
Absent pulmonary valve cusps, absent ductus arteriosus, and right aortic arch were determined on the 3VT view

**Figure 9:**
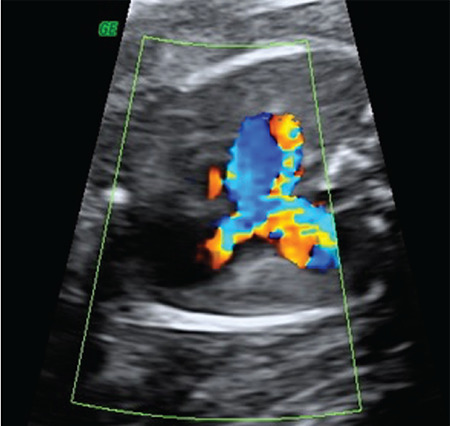
Increased peak flow velocity through the pulmonary valve annulus and a retrograde diastolic flow originating from the pulmonary valve annulus was revealed by color Doppler ultrasound

All four patients with a prenatal diagnosis of PA anomalies with or without cardiac malformations were reviewed for intrauterine course and outcome between 2018 and 2020. The mean gestational age at the time of admission was 24.8 (range, 22-28) weeks and the mean maternal age was 32.5 (range, 30-35) years. Three of four patients were referred to our center due to suspicion of congenital heart disease, and the remaining patient was detected during a routine ultrasonographic examination. All cases were singleton pregnancies and no extracardiac anomalies were identified. The four cases of the PA anomalies were LPA sling, LPA originating from the ascending aorta, DA agenesis with right aortic arch - TOF, and DA agenesis with absent pulmonary valve - TOF, respectively. Only one of the four patients accepted an invasive procedure and the results of chromosome analysis and FISH analyses for microdeletion 22q11.2 were normal. Three of four fetuses were delivered by cesarean section (CS) due to previous CS at term. A physical examination of the other three infants was performed and no dysmorphic findings were found in the postnatal period. Three infants except for one with DA agenesis and accompanying absent pulmonary valve - TOF, were clinically stable and they did not require surgery at the time of writing. A summary of the data from all four patients is shown in the Table and each clinical case report is described below and accompanied by supporting figures (Figures 1, 2, 3, 4, 5, 6, 7, 8, 9).

### Case 1

A gravida (G) 5, parity (P) 1, abortus (Ab) 3, 33-year-old woman with a non-consanguineous marriage, underwent routine ultrasound examination at 22 weeks gestation and fetal echocardiography (Samsung Ultrasound H60) was performed for suspected cardiovascular malformation. The family history was not significant regarding congenital diseases or malformations. Prenatal echocardiography demonstrated that the LPA originated from the right PA and then, turning left, crossed midline behind the trachea, anterior esophagus, and descending aorta towards the hilum of the left lung (Figure 1). In the 3VT view, the trachea was encircled by the aberrant LPA and left-sided DA. The diameter of right and left PAs was equal and the thymus size was in the normal range for 22 weeks gestation. Neither congenital anomalies and polyhydramnios nor genetic abnormalities including abnormal karyotype and Di George syndrome were detected in amniocentesis. Due to placenta previa totalis, a 3480 g female baby was delivered by CS in a tertiary care center at 38 weeks of gestation. The APGAR scores were 9 and 10 at 1 and 5 minutes, respectively. The diagnosis of LPA sling was confirmed using postnatal echocardiography on the first day after birth, and the peak systolic velocity of the left pulmonary was measured in the normal range, excluding the stenosis or hypoplasia of LPA. Postnatal axial contrast-enhanced CT confirmed the aberrant LPA and normal-appearing lungs and tracheobronchial anatomy (Figure 2). The asymptomatic baby was discharged from hospital on the third day after birth and she has been still symptom-free for eight months at the time of the writing.

### Case 2

A G2P1 32-year-old woman with a history of hyperthyroidism was referred to our perinatology clinic for second trimester ultrasonographic and fetal echocardiographic screening at 26-weeks gestation. The family history was negative for congenital heart defects, chromosomal abnormalities, or unexpected newborn death shortly after birth. There were no other malformations outside the heart of the fetus (Samsung Ultrasound H60). Fetal echocardiography showed that visceroatrial situs solitus with normal systemic and pulmonary venous connections and normal four-chamber view. Fetal cardiac findings were as follows: the aortic arch and ductal arch were located on the right side of the trachea and four vessels on the 3VT view (Figure 3). Although the right PA arose from the pulmonary trunk, the origin of the LPA could not be seen clearly. To evaluate the anatomy in the transverse plane, Doppler ultrasonography was used, sweeping from inferior to superior and tracking the LPA from the hilum of the left lung, and anterior the trachea revealed its origin from around the vicinity of the ascending aorta closed to the brachiocephalic artery (Figure 4). The thymus was seen normally behind sternum at the level of the 3 vessels trachea view. The parents were consulted for genetic diseases but cordocentesis for karyotyping and analysis of 22q11 deletion was refused by the patient. At 40 weeks of gestation, a female infant of 3,565 g was born after an uncomplicated vaginal delivery with APGAR scores of 9 and 10 at 1 and 5 minutes, respectively. The physical examination of the baby was normal without any dysmorphic features and arterial oxygen saturation was normal. Neonatal echocardiography revealed LPA origin in the posterior aspect of ascending aorta, midsegment stenosis in the LPA with a peak pressure gradient of 30 mm Hg. Right-sided aortic arch, right-sided patent ductal arteriosus, and mild tricuspid regurgitation were seen in the postnatal echocardiography. Two days after birth, we performed CT angiography and confirmed the fetal diagnosis showing the described left pulmonary hemitruncus. The newborn was discharged at 3 days after birth without cardiac or respiratory symptoms. The baby was doing clinically well in her last clinic visit at 4 months of age.

### Case 3

A 35-year-old G4P1Ab2 refugee woman was referred to our perinatology clinic due to a suspected a fetal heart defect. There was no family history of congenital heart disease nor did she have any risk factors leading to congenital heart disease. The results of a fetal echocardiogram at 23 weeks of gestation showed the right aortic arch, malalignment ventricular septal defect, and overriding aorta in five-chamber view (Figure 5). Further, DA could not be seen arising from the main PA and connecting to the descending aorta in the 3VT view and sagittal view, respectively (Samsung Ultrasound H60). Pulmonary valve, main PA, and the branches of the pulmonary valve were in the normal range. The diameters of the pulmonary annulus, right and LPA were 2.49 mm, 1.55 mm, and 1.87 mm, respectively (Figure 6). Color Doppler confirmed the presence of an overriding aorta with blood draining from both ventricles into the aorta. The antegrade flow was seen in the main and branch of the PA but DA could not be demonstrated using color Doppler. After a prenatal genetic consultation, amniocentesis for karyotyping and analysis of 22q11 deletion was declined by the parents. The female fetus was delivered by CS due to previous CS at 38 weeks gestation. The neonate did not need any cardiac or respiratory support and weighed 3175 g. There were no findings suggesting any genetic anomaly in the phenotype of the newborn baby. A postnatal transthoracic echocardiogram revealed a normal four-chamber view; however, the aortic root was slightly shifted in the right ventricle and located to the right of the trachea, DA was absent as in the fetal findings. The diameters of the pulmonary annulus, right and LPA were 5.8 mm, 4.5 mm, and 4.8 mm, respectively. The peak pressure gradient at the level of the pulmonary valve was approximately 23 mm Hg at discharge from the hospital. She has remained uneventful for two months at the time of the writing. Surgical correction is delayed until she reaches the appropriate age.

### Case 4

A G2P1 30-year-old woman was sent to our clinic with a suspicion of congenital diaphragmatic hernia on obstetrical ultrasound at 28 weeks of gestation. The healthy pregnant women had no genetic disorders or fetal heart anomaly in her family history. Anatomic screening was normal except for the fetal heart (Samsung Ultrasound H60). The fetus presented with situs solitus, levocardia, and the atrioventricular connection were normal. A slightly larger right heart was shown on a four-chamber view; the pulmonary valve annulus was hypoplastic (4.5 mm); a subaortic ventricular septal defect with the overriding aorta, severe dilatation of the pulmonary trunk and its branches  (right and LPA were 1.2 mm and 13.8 mm, respectively) were seen on the outflow tract of the right ventricular view (Figure 7); absent pulmonary valve cusps, absent DA, and right aortic arch were determined on the 3VT view (Figure 8). Increased peak flow velocity through the pulmonary valve annulus and a retrograde diastolic flow originating from the pulmonary valve annulus was revealed using color Doppler ultrasound (Figure 9). Absent pulmonary valve with TOF was diagnosed based on the following findings: absent pulmonary valve, absent DA, right aortic arch, presence of malalignment ventricular septal defect, and an overriding aorta. The main PA diameter was 5.63 mm, and the left and right artery diameters were 3.37 mm and 3.33 mm, respectively. The mother refused to undergo karyotype analysis and FISH analysis to detect the 22q11 microdeletion. Fetal hydrops did not develop with compensatory extension of the left and right PA and with the shunt through a patent foramen ovale during the follow-up from the 28 weeks of gestation to the delivery. A male fetus was born by repeat CS at 39 weeks of gestation. Postnatally, transthoracic echocardiographic findings were consistent with the prenatal diagnosis. Cardiac results included TOF, aneurysmal dilatation of PAs branches, and the right ventricle was slightly enlarged. When the baby presented early with airway compression from aneurysmal PAs, early correction surgery was performed successfully at age 2 months.

## Discussion

Reports of prenatal diagnosis of PA malformations, including agenesis of the DA, absent pulmonary valve, abnormal branching of pulmonary vessels, and origin of the PA, are extremely rare anomalies^(1,6)^. PA anomalies may be isolated or accompanied by other complex cardiac and extracardiac anomalies. Surgical treatment and prognosis are also variables because the pathologic anatomy is very different^(2)^. We described four cases of the PA anomalies that were identified in fetal echocardiography and confirmed in the newborn period using echocardiography and CT angiography (Table 1).

The PA derives from the right ventricle, courses towards the left of the more posterior ascending aorta, and branches after a short course in the normal manner. The first branch is the right PA, left branch subsequently. The PA continues distally towards the left side and into the DA, which connects to the descending aorta in anatomically normal fetuses. In healthy fetuses, ductal and transverse aortic arches merge with a course to the left of the trachea and no vascular structures exist posterior or surround the trachea at the level of three-vessel tracheal view, which is the most important view in the diagnosis of conotruncal abnormalities^(7)^. During the normal development of the cardiovascular system, the aortic arches develop from the aortic sac, with a pair of branches (right and left), and initially, six pairs of aortic arches are present and symmetrical. They develop and regress at different stages of development. In the process of normal embryogenesis, the sixth aortic arches separate into ventral and dorsal segments and the ventral part of the arches forms the proximal branch of the PAs bilaterally. The left ventral arch is responsible for the formation of the pulmonary trunk, the left dorsal sixth arc continues as the DA and connects the main pulmonary trunk with the left dorsal aorta^(8)^. The LPA originates as a branch of the left ventral sixth aortic arch and capillaries that arise from the pulmonary postbranchial plexus surrounding the lung bud by the 8^th^ week of embryonic development^(8)^.

The first prenatal diagnosis of LPA sling was demonstrated in 2011 during a fetal ultrasound examination at 32 weeks gestation by Yorioka^(9)^. To date, a few cases of prenatally diagnosed LPA sling have been reported^(10)^. For the development of aberrant LPA, it was proposed that if the connection between the left lung bud ventrally and left six arches fails, the connection occurs between right six aortic arch and the developing left lung bud dorsally, resulting in a LPA forming from the right PA, passing posterior to the trachea^(11)^. We described one prenatal case with LPA sling with follow-up from 22 weeks gestation to a postnatal age of 2 years. After birth, axial contrast CT and postnatal echocardiography findings confirmed the prenatal diagnosis of LPA sling. According to the Wells classification of LPA sling, CT demonstrated that the bronchial anatomy was type 1A, the LPA arose from the right PA, and coursed posteriorly between the trachea and esophagus^(12)^. The carina was typically located at the T4-T5 level and the aberrant LPA did not cause compression of the trachea, bronchus, and esophagus. Symptoms of this condition usually manifest clinically in the neonatal period in 50% of the patients, and in 65% they occur before 1 month of age^(13)^. The most common symptoms are related to the respiratory system, causing dyspnea wheezing and stridor. The symptoms are produced by compression of the tracheobronchial tree and esophagus, causing tracheobronchial stenosis, tracheomalacia, and airway compression in the postpartum period. About half of all cases are associated with other cardiac and non-cardiac defects such as atrial septal defect, persistence left superior vena cava, ventricular septal defect, abnormal pulmonary lobulations, and tracheal bronchus^(13)^. Morbidity and mortality often depend primarily on the presence of tracheobronchial tree anomaly and especially tracheal or bronchial stenosis other than LPA sling, with mortality rates as high as 50% without surgical correction^(11)^. Although half of all patients have symptoms, in our case, LPA sling was an isolated anomaly without symptoms at the time of writing.

Anomalous origin of unilateral PA is a rare congenital pulmonary vascular malformation, with one branch of the PA originating from the ascending aorta and the other arising from the main PA. The embryogenesis and development of DA and the aortic arch is still uncertain. DA originates from the left sixth arch, presumably, and DA is connected with the first part of the LPA. As the 6^th^ arches do not develop partially or completely on the left side, the LPA cannot attach to the main PA and the aortic sac persists from which the PA originates^(14)^. Right hemitruncus is more frequent than left hemitruncus^(14)^: however, left hemitruncus is more associated with other cardiovascular anomalies with either TOF or right aortic arch^(15)^. After birth, pulmonary vascular resistance decreases progressively in healthy babies; however, excessive blood flow into the anomalous origin of unilateral PA from the aortic arch leads to pulmonary over circulation and the affected lung is exposed to systemic pressure, whereas the other lung received the entire blood supply volume from the right ventricle. As a result of pulmonary over circulation, pulmonary hypertension, subsequently, and heart failure develops. To avoid irreversible pulmonary vascular damage and the development of pulmonary hypertension, early diagnosis and surgical correction are crucial in hemitruncus^(16)^. It has been reported that mortality rates are very high in patients who are not surgically treated^(14)^. Prenatal detection of hemitruncus is crucial in fetal echocardiography because early surgical correction improves survival^(17)^. In this case report, we documented the prenatal diagnosis of a patient with left hemitruncus, right-sided aortic arch, and right-sided ductal arch in the 3VT view. Postnatal echocardiography and axial contrast-enhanced CT confirmed the fetal diagnosis showing the described left pulmonary hemitruncus, and midsegment stenosis in the LPA with a peak pressure gradient of 30 mm Hg was determined, which was confirmed by cardiac catheterization. Detection of the fourth vessel in the 3VT should lead to a search for the differential diagnosis including abnormal pulmonary venous connections, persistent left superior vena cava, esophageal or bronchial tree anomalies, or cystic thoracic mass. We emphasized that left hemitruncus should be taken into consideration in differential diagnosis and the importance of demonstrating branching of both PAs.

In the normal fetal circulation, DA carries 78% of the right ventricular blood away from the lungs and joins the descending aorta to supply the lower part of the fetus. The underlying mechanism of DA agenesis is not yet clear. One of the mechanisms is agenesis of DA occurring unless the left dorsal sixth arc continues as the DA. The secondary mechanism is where blood in the right ventricle, which has high oxygen saturation, cannot be transported to the lungs due to the severe obstruction of the PA and the higher oxygen saturated aortic blood diverts from aorta to the DA, thereby inducing constriction^(18)^. Embryologically, the right aortic arch occurs due to the persistence of the right dorsal aorta instead of the left dorsal aorta. The embryologic process causing the development of TOF is not known; it is assumed that an anterior and cephalad deviation of the infundibular septum results in a malalignment ventricular septal defect, and the aortic root overrides the ventricular septal defect, causing a subsequent right ventricular outflow obstruction. Absent Pulmonary Valve syndrome may be accompanied by a ventricular septal defect called TOF with absent pulmonary valve, and this is generally associated with the absence of the DA. However, if the ventricular septal defect does not occur, DA is usually present. In the absence of DA, the main pulmonary trunk cannot connect the left dorsal aorta, PA blood pressure increases, severe pulmonary regurgitation develops, resulting in an enlarged PA, and this pathologic process interferes with the normal development of the pulmonary valve. Isolated agenesis or premature closure of the DA is an uncommon congenital cardiac malformation; they are usually associated with TOF, absent pulmonary valve, truncus arteriosus, or maternal use of prostaglandin synthetase inhibitors.

We demonstrated two cases of agenesis of DA and the first was accompanied by TOF and right aortic arch with a normal pulmonary valve at 23 weeks of gestation, the second presented with absent pulmonary valve with TOF. Although DA did not develop in the first case, pulmonary valve and branches of the PA were seen in the normal range because blood from the right ventricle to the aorta exited via a malalignment ventricular septal defect and pulmonary valve, and branches of the PA were not exposed to the high pressure of right ventricle. In TOF, agenesis of a DA would probably occur at the beginning of the pregnancy; patent foramen ovale and large ventricular septal defect would be able to carry the blood from the right ventricle into the descending aorta, thus, the pulmonary valve, pulmonary vascular bed, and PA branches could develop normally^(19)^. The component of absent Pulmonary Valve syndrome includes the absence or rudimentary pulmonary valve, stenosis of the pulmonary valve annulus, dilatation of the pulmonary trunk, and left and right branches of PAs. This condition is rarely isolated, it is most often associated with TOF and agenesis of DA. The fetal echocardiographic diagnosis was confirmed for both cases. The first patient is 4 months old at the time of writing and no therapeutic intervention has been performed yet. In the second case, early successful correction was performed due to significant airway compression when the baby was aged 2 months.

Fetal echocardiography is considered to be an important, non-invasive, and safe diagnostic modality for assessing congenital heart anomalies. Our cases highlight the variability of abnormalities of the PA. Although outflow tract anomalies have a characteristic echocardiographic appearance in the 3VT view, prenatal diagnosis of these anomalies may be missed or anomalies may be misdiagnosed, especially abnormal branching of the PA^(20)^. Therefore, the detection of pulmonary anomalies prenatally may be challenging but is very important because postnatal careful follow-up of the neonate improves short and long-term outcomes. When the pulmonary branch cannot be displayed as a Y shape, it is vital to trace the course of left and right PA branch to diagnose the anomaly.

## Conclusion

Branching of the PA, presence of DA, location of the aortic, and ductal arch should be routinely screened in the prenatal anatomic examination and the 3VT view determines primary clues of PA malformations. Physicians should increase their awareness in the prenatal diagnosis of PA anomalies to avoid unfavorable outcomes and the 3VT view needs to be performed excellently during routine fetal anatomic examinations.

## Figures and Tables

**Table 1:**
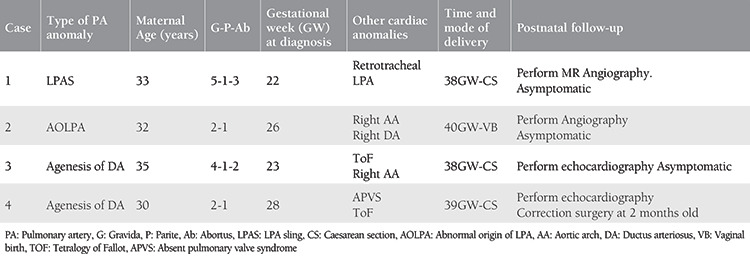
Summary of cases with pulmonary artery anomalies
